# In focus: perplexing increase of urinary stone disease in children, adolescent and young adult women and its economic impact

**DOI:** 10.3389/fmed.2023.1272900

**Published:** 2023-10-23

**Authors:** Guido Filler, Sumit Dave, Victor Ritter, Sherry Ross, Davis Viprakasit, Joseph E. Hatch, Jennifer Bjazevic, Jeremy Burton, Donna Gilleskie, Jason Gilliland, Feng-Chang Lin, Nina Jain, J. Andrew McClure, Hassan Razvi, Vipin Bhayana, Peter Wang, Sherry Coulson, Nabil Sultan, John Denstedt, Loretta Fearrington, Maria E. Diaz-Gonzalez de Ferris

**Affiliations:** ^1^Department of Paediatrics, Western University, London, ON, Canada; ^2^Department of Medicine, Western University, London, ON, Canada; ^3^Department of Pathology and Laboratory Medicine, Western University, London, ON, Canada; ^4^Department of Surgery, Western University, London, ON, Canada; ^5^Department of Biostatistics, University of North Carolina at Chapel Hill, Chapel Hill, NC, United States; ^6^Department of Urology, University of North Carolina at Chapel Hill, Chapel Hill, NC, United States; ^7^Department of Pediatrics, University of North Carolina at Chapel Hill, Chapel Hill, NC, United States; ^8^Department of Microbiology and Immunology, Western University, London, ON, Canada; ^9^Department of Economics, University of North Carolina at Chapel Hill, Chapel Hill, NC, United States; ^10^Department of Geography, Western University, London, ON, Canada; ^11^North Carolina Translational and Clinical Sciences Institute, University of North Carolina at Chapel Hill, Chapel Hill, NC, United States

**Keywords:** urinary stone disease, sex differences, urinary phthalates, population-based epidemiology, obesity, diet, hormones, environmental factors

## Abstract

**Background:**

Urinary stone disease (USD) historically has affected older men, but studies suggest recent increases in women, leading to a near identical sex incidence ratio. USD incidence has doubled every 10 years, with disproportionate increases amongst children, adolescent, and young adult (AYA) women. USD stone composition in women is frequently apatite (calcium phosphate), which forms in a higher urine pH, low urinary citrate, and an abundance of urinary uric acid, while men produce more calcium oxalate stones. The reasons for this epidemiological trend are unknown.

**Methods:**

This perspective presents the extent of USD with data from a Canadian Province and a North American institution, explanations for these findings and offers potential solutions to decrease this trend. We describe the economic impact of USD.

**Findings:**

There was a significant increase of 46% in overall surgical interventions for USD in Ontario. The incidence rose from 47.0/100,000 in 2002 to 68.7/100,000 population in 2016. In a single United States institution, the overall USD annual unique patient count rose from 10,612 to 17,706 from 2015 to 2019, and the proportion of women with USD was much higher than expected. In the 10–17-year-old patients, 50.1% were girls; with 57.5% in the 18–34 age group and 53.6% in the 35–44 age group. The roles of obesity, diet, hormones, environmental factors, infections, and antibiotics, as well as the economic impact, are discussed.

**Interpretation:**

We confirm the significant increase in USD among women. We offer potential explanations for this sex disparity, including microbiological and pathophysiological aspects. We also outline innovative solutions – that may require steps beyond typical preventive and treatment recommendations.

## Introduction

Urinary stone disease (USD) can result in extreme pain, hospitalizations, and costly/invasive surgical procedures. USD is a public health concern, as it is associated with osteoporosis, the development of chronic kidney disease, and shortened life-expectancy (1). We are witnessing an unprecedented increase of USD, in adolescents and young adults (AYA), especially amongst women (2), for unclear reasons. Historically, women were at lower risk for USD, thought to be due to higher urinary citrate (a protective factor against USD) and differences in uric acid metabolism (3). There is a paucity of studies on USD recurrence in younger populations, but Novak et al., reported a men to women ratio of 1:2.3 in 2003 (4).

This viewpoint describes current USD epidemiologic trends by sex, highlights two North American experiences, describes the economic impact of this condition, offers potential pathophysiological explanations for the rising incidence of USD in women, and suggests innovative solutions targeting specific etiopathological pathways to modify these trends.

### Epidemiology of urinary stone disease by sex

**Figure 1:**
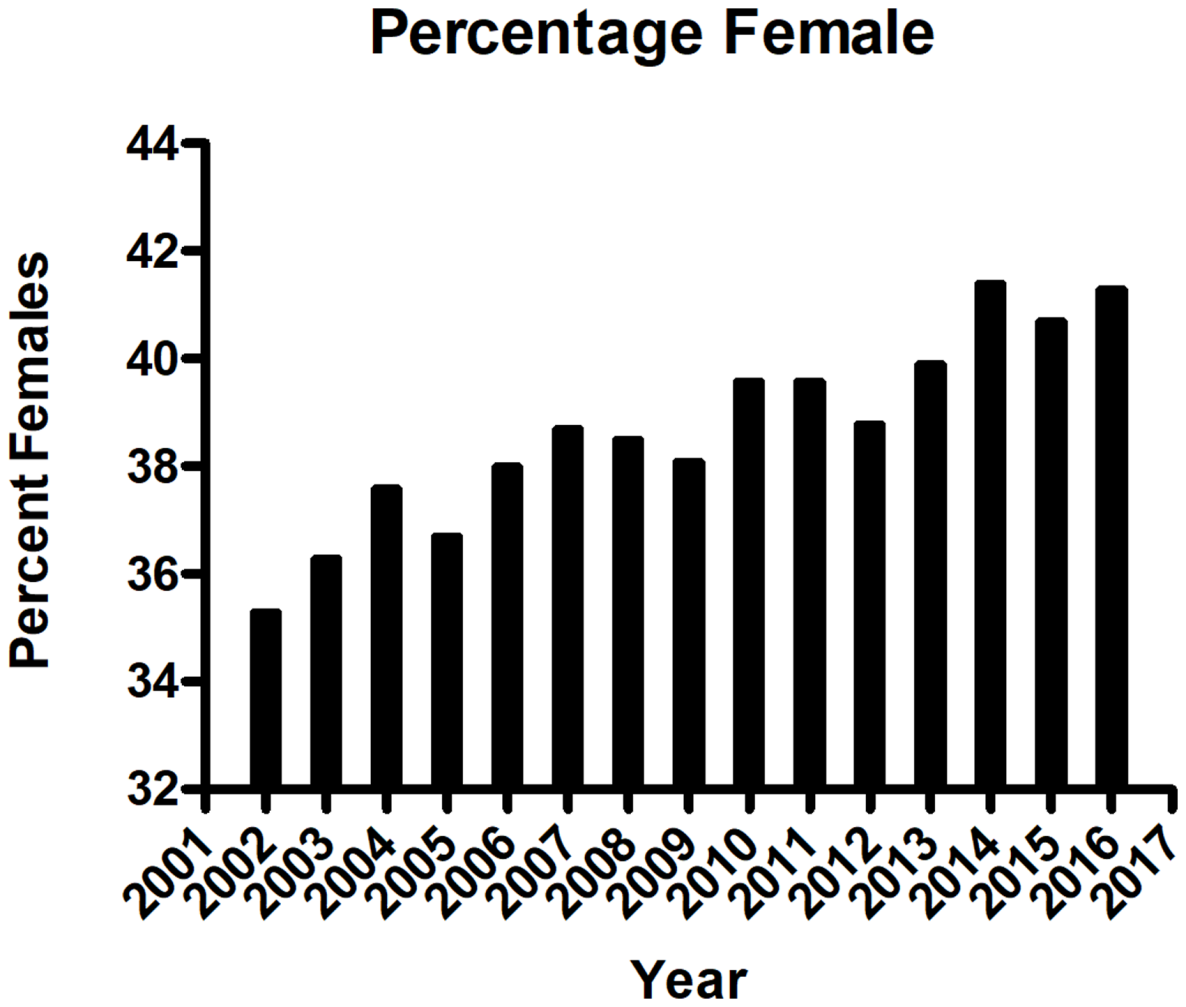
Sex of patients with urinary stone disease requiring surgery in the province of Ontario, Canada, per year between 2002 and 2016, *p* < 0.001, ANOVA.

In the United States, the incidence of USD has increased to 1 in 11 people (11% of men, 5.6% of women) (5). Historically, USD affected older men (5) who typically produce calcium oxalate stones; however, this condition is occurring in younger populations (2), particularly women who produce predominately apatite stones (calcium phosphate) and produce more alkaline urine (6). Tasian et al. described in an epidemiological study in South Carolina between 1997 and 2012 that the greatest increase of USD was observed among 15–19 year olds (7). After adjustment for age and race, the increase occurred among females while the rates in males remained constant (7). To corroborate these findings, we conducted a population-based study in the province of Ontario, Canada from 2002 to 2016, examining 102,772 primary USD surgical cases. There was a significant sustained annual increase of USD-related surgical interventions from 5,678 in 2002 to 9,234 in 2016 (a 46% increase overall). During this same time-period, the Ontario population increased only 12.5%. The incidence rose from 47.0/100,000 in 2002 to 68.7/100,000 population in 2016. Additionally, the average age of first-time USD surgery patients decreased over time, and the proportion of female patients significantly rose from 35% (1977 women and 3,619 men in 2002) to 42% (3,794 women and 5,395 men in 2016); *p* < 0.0001 (ANOVA; Figure 1). Fortunately, while the population of patients <18 years of age slightly decreased over this time, there was no significant change in the number of surgical interventions for USD (8). Mean pediatric patient age was 11.27 ± 5.70 years with a median of 14.00 years, and 51.2% were female. In 2002, 44.05% of the pediatric patients were female, and this percentage gradually increased to 62.90% in 2018, *p* < 0.001 (8). Rural residence was observed in 12.8%. We do not know what the total number of patients with USD was during the same period; however, in a review from 20 countries, the unadjusted incidence of USD patients needing surgery was 206.6/100,000 (2), while the incidence of symptomatic USD was 1,116/100,000 (9), suggesting that 18% of USD patients require surgery. This finding aggregates to more than ½ million people who had at least one episode of USD over this 15-year period in the province of Ontario.

**Figure 2:**
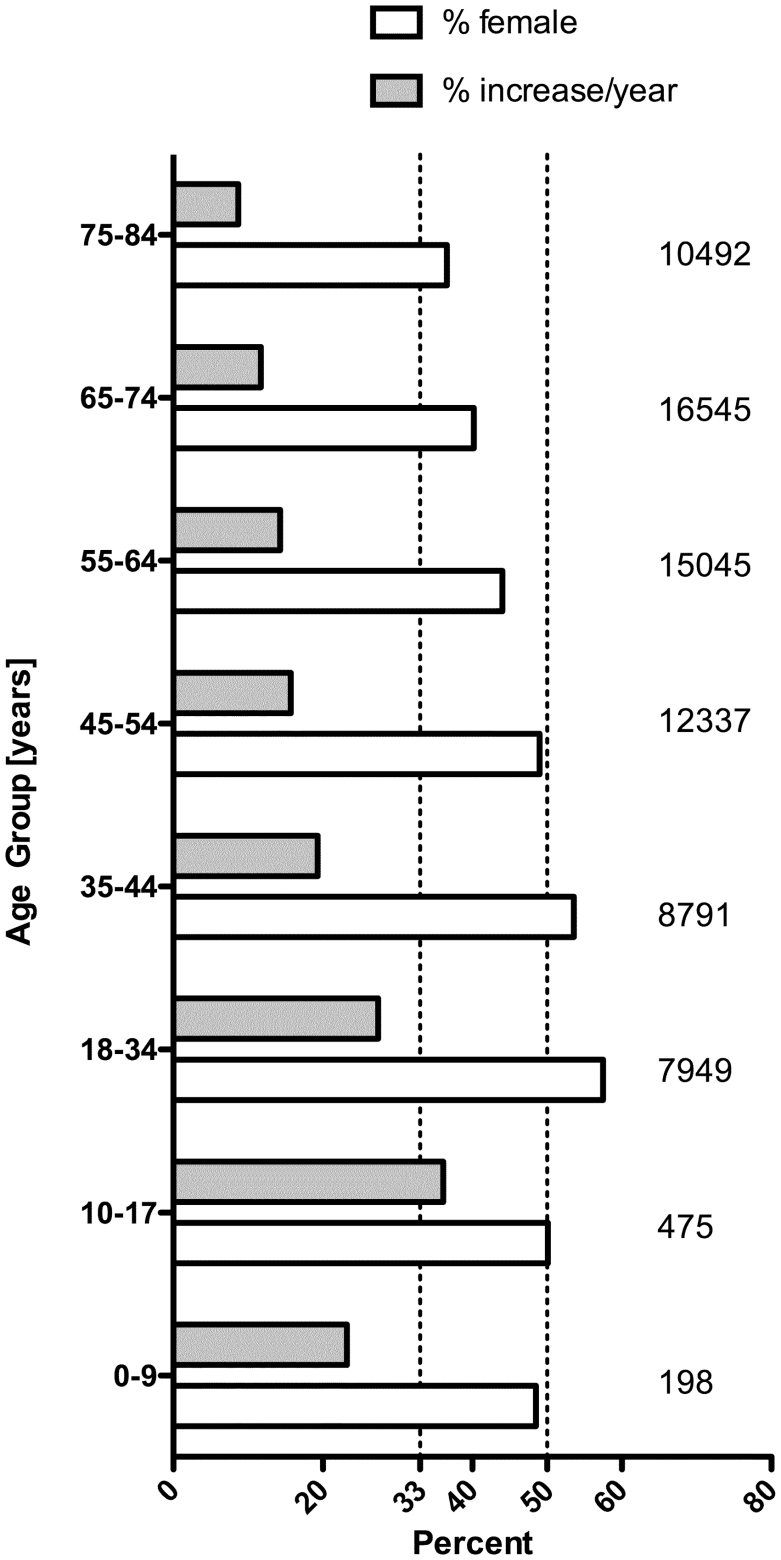
Average annual percent increase and percent women among 75,275 unique new urinary stone disease patients at UNC between the years 2015 and 2019. On the right side, the number of cases in each age group are given. There was no significant increase among patients >85 years old (data not shown).

Data from the 11 hospitals of University of North Carolina Health Care System in the United States, a warmer climate area than Ontario, Canada, showed a similar increase in trends for all USD cases from 2015 to 2019 not just for those who needed surgical intervention. The overall USD annual unique patient count rose from 10,612 to 17,706, and the proportion of women with USD was much higher than the traditional sex distribution. In the 10–17-year-old patients, 50.1% were girls; with 57.5% in the 18–34 age group and 53.6% in the 35–44 age group. Figure 2 demonstrates a significant increase in USD cases (over just 4 years), especially in the younger patients. The reasons for this perplexing finding have not been elucidated. These patterns are particularly troubling as USD is a chronic/recurrent condition with 50% of patients having recurring stones within 3 years (10). These data are in keeping with Carmen Tong’s recent review that declared pediatric USD to be a public health and economic burden in the United States (11).

## Potential explanations for the sex disparity in urinary stone disease

**Figure 3:**
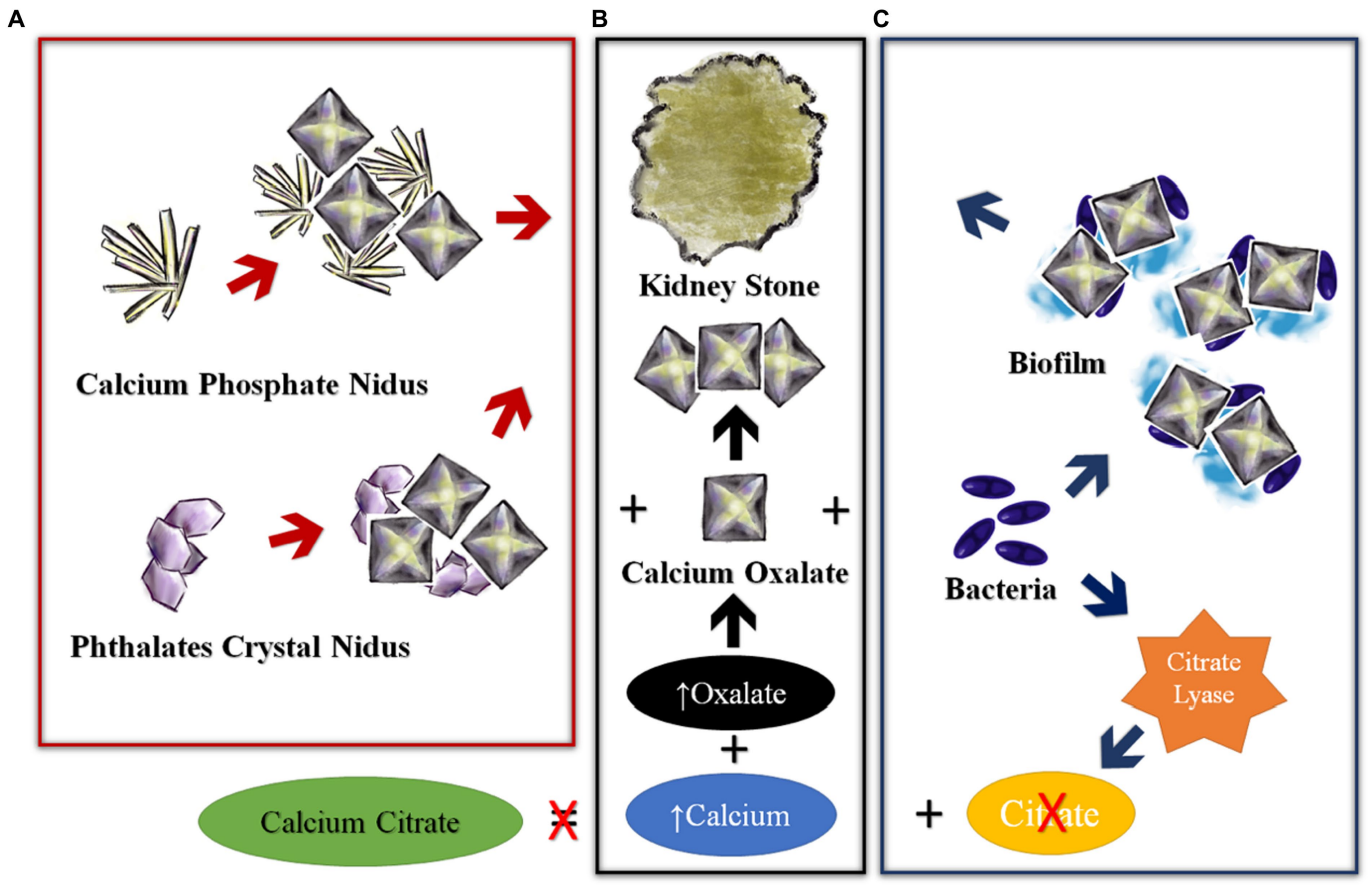
Simplified concepts of kidney stone formation. **(A)** Nidus formation in the loop of Henle, **(B)** Stone formation, **(C)** Modification in urinary tract.

The exact risk factors of USD and kidney stone formation process are not completely understood (12). The current hypothesis for USD is based on several steps that involve the presence of a nidus and conditions in the milieu of the urine that favor crystallization. A simplified scheme is provided in Figure 3, in which crystals of calcium oxalate or calcium phosphate (or potentially other crystals/substances such as uric acid or phthalates) form the initial nidus, seeding initial stone formation. As reviewed by Gay et al., the exact mechanisms of nidus and eventual stone formation are unknown, but there are several types of stones or nidus-crystals recovered from patients with USD suggesting several mechanisms may be involved (13). Importantly, bacteria are commonly detected in kidney stones, demonstrating that there are critical pathways of interaction between substrates in the urine, the urinary microbiome and biofilm formation, which ultimately result in stone formation (Figure 3). Multiple factors may also play a role, namely obesity, diet, hormones, environmental factors/pollutants, infectious agents, and antibiotic use (especially for sexually transmitted diseases).

### Obesity

The worldwide prevalence of obesity has nearly tripled between 1975 and 2016. The prevalence of overweight or obesity amongst 5–19-year-olds has risen from 4% in 1975 to 18% in 2016, similarly among boys and girls where 18% of girls and 19% of boys were overweight. Studies have associated obesity as a risk factor for USD, with lower urine pH, especially if women have low urine pH (14). Obesity is also associated with higher testosterone, which may be related to USD in 25–50 year-old women (15). Obese women have greater intestinal oxalate absorption, which is a well-known risk factor for USD (16). However, USD in pediatric patients has not been found to be associated with obesity (17, 18).

### Diet

The steady increase in dietary sodium intake in Western countries (typically >3,500 mg/day, whereas the WHO recommends <2000 mg/day), has been associated with USD (19). Increased salt intake is perhaps the single most amenable lithogenic risk factor, as there is a direct relationship between urinary sodium excretion and urinary calcium wasting (12). Additionally, dietary fructose (significantly increased in the Western diet since the early 1980’s) may be associated with increased levels of uric acid, oxalate, decreased magnesium and pH in the urine as well as mildly increased PTH, and decrease in serum ionized calcium in serum. This milieu promotes USD in a pediatric mouse model (20). High salt intake and animal protein, are major risk factors for USD; however, dietary habits have changed much slower than the observed increases in the USD cases and experts agree that changes in diet alone do not explain the rapid increases in USD. Unfortunately, socioeconomic status is inversely correlated with salt intake as low cost foods are highly salted and up to 75% of the salt intake is due to processed food (21). Similarly, groups with low income and education are vulnerable to high sugar diets (22). Uric acid crystals could create a nidus as well, and these are formed after consumption of high fructose corn syrup in mice (20). This potential relationship is depicted in Figure 3. A uric acid nidus might explain the high prevalence of apatite stones in AYA women with USD (23). However, such a relationship has only been hypothesized (24), but not proven in pediatric USD patients. Also, rather than fructose itself, high fructose corn syrup, which is the industry standard sweetener for soda and many foods, has to be considered as a potential risk factor (25, 26). Using high-resolution transmission electron microscopy, Gao et al. have shown that both nano-uric acid and nano-calcium phosphate may serve as nidus for USD formation (27).

### Hormones

Pregnancy induces hormonal and physiological changes that may induce stone formation. Increased intestinal calcium absorption is mediated by increases in parathyroid hormone-related protein, estradiol, prolactin and placental lactogen which all stimulate increase production of 1,25 dihydroxy Vitamin D (28). Hypercalciuria, hyperoxaluria and uricosuria are related to increases in glomerular filtration rate (GFR) during pregnancy (29). Also, urinary pH becomes more alkaline during pregnancy, increasing calcium phosphate supersaturation (12). Thongprayoon et al. have shown that pregnancy increases the risk for first-time USD (30). NHANES reported a 2-fold increase in stone risk among women who had previously been pregnant compared to age-matched controls who had never been pregnant (31). However, pregnancy rates have not increased significantly since the 1970s and therefore do not explain the increasing rate of USD among women. Oral contraceptives have been linked to increased bone turnover and a potential risk of osteoporosis and USD (28).

Another theory is the increasing rate of polycystic ovary syndrome (PCOS), prevalent in 6–10% of women and associated with hyperandrogenism, as a risk factor for USD (32). PCOS is associated with insulin-resistance and increased risk factor of impaired glucose tolerance, dyslipidemia and non-alcoholic fatty liver disease, all associated with USD (33). Little is known about the potential contribution to stone risk based on normal hormonal changes that occur during puberty. Studies on pubertal females have been elusive.

### Environmental factors/pollutants

It has been hypothesized that behavioral and environmental factors such as ambient temperature, changing patterns of infection (with expanded use of antibiotics) (34), exposures to environmental toxins and microplastics such as phthalates (35), may be contributing to the increasing incidence of USD. Phthalates (multifunctional chemicals used in a wide variety of plastic products) get into the body through many routes including ingestion of drinking water. Phthalate metabolites are excreted in the urine and could form a nidus for USD, though there is currently no evidence for this. The metabolite monobutylphthalate is excreted in urine at three-fold higher concentrations in women aged 20–40, and similar to oxalate, it is practically insoluble (36). One might argue that dietary factors change faster than environmental factors; however, microplastic exposure is increasing at alarming rate. They have been detected in many marine species, but also in drinking water and in numerous foods, such as salt, honey, and marine organisms. Exposure to microplastics can also occur through inhaled air (37).

Urinary polyaromatic hydrocarbons, a class of chemicals that occur naturally in coal, crude oil, and gasoline, and result from burning coal, oil, gas, wood, garbage, and tobacco, have also been associated with USD (38), although it must be stressed that an association does not infer causality.

The impact of climate and climate change (global warming) on USD have been previously reported (39). Brikowski et al. (40) and Kaufman et al. (41) have studied this. The latter study is interesting as it also modeled a total excess cost in the state of South Carolina of up $99 million (41).

### Infectious factors/antibiotics

While the exact incidence is difficult to determine, urinary tract infections (UTIs) are very common. UTIs with urease-producing organisms initiate the formation of struvite (magnesium ammonium phosphate) and apatite (calcium phosphate) calculi. Furthermore, there has been a significant increase of sexually transmitted chlamydia infection. Typical treatments for chlamydial infection involve Doxycycline or Azithromycin, which may reduce *Oxalobacter formigines* in the gut, increasing oxalate absorption and resulting in oxaluria. Sulfamethoxazole, Cephalosporins, Fluoroquinolones and Nitrofurantoin are also associated with USD (34).

Our metagenomic study of adults with USD (*n =* 83) compared to controls (*n =* 30) found that those with USD had significant evidence of chemical/antibiotic microbiota exposures with reduced microbial genetic capacity to produce vitamin B6 (a protective factor against oxalate stones), amongst several other microbiota-encoded metabolic abnormalities linked to this condition (34). A metagenomic pediatric study of fecal samples suggests that those with USD have decreased proportions of bacteria-produced butyrate, a short chain fatty acid important for enterocytes to prevent increased intestinal permeability and increased oxalate absorption (42).

### Economic impact of USD

In adults, USD causes a great financial burden with mean annual healthcare expenditures more than double the expenditures of those without this condition ($6,532 vs. $3,038/person/year) (2). However, this figure ignores the lifetime implications of this condition in AYA, which often becomes chronic, and the impacts of other diseases such as osteoporosis and chronic kidney disease, which are strongly associated with USD. The overall cost of diagnosis, treatment and prevention of USD was estimated to be $4 billion dollars in the United States in 2007 and is projected to increase by more than $780 million by 2030 (43). Wang et al. reported the median cost of inpatient care among pediatric USD patients in 2015 at $13,922 in charges per admission (44). Sturgis et al. analyzed a contemporary United States cohort of pediatric USD patients diagnosed between 2011 and 2018 with an overall cost of $117.1 million US dollars for just over 10,000 patients (45). However, given the epidemiological trends listed here, that increase may be much higher. These figures only account for the medical management and do not include the impact on productivity losses, transportation costs, short-term care needs, etc.

It is imperative to quantify the direct and indirect economic effects of the chronic health impacts of USD, given increasing evidence that USD is rising in children. Direct costs include medical visits, hospitalizations, and pharmaceutical treatment. Indirect costs include impacts on educational attainment (i.e., graduation from high school, college attendance, and performance), which may be impacted by periodic painful episodes or surgical interventions that reduce academic performance or attendance. These indirect costs continue beyond schooling age and include similar productivity impacts in the workplace such as absenteeism and presenteeism (i.e., lower on-the-job productivity). Peer-reviewed research on the effect of chronic illness on absence and presenteeism demonstrates a range of 2–3 times as much lost time from presenteeism as from absence (46).

The costs to the employer include wage replacement, non-wage compensation, and the hiring of temporary workers or use of overtime to fill voids. Data from nationally representative registries may assist predicting the indirect costs of USD and comorbidities on work productivity. Wages may not be a sufficient measure of lost productivity (but would proxy for time costs associated with transportation or care needs). For example, Nicholson et al. estimated wage “multipliers” for 35 different jobs, where the multiplier is defined as the cost of an absence to the business, as a proportion of the absent worker’s daily wage (47). Measurements of necessary variables, such as instruments for measuring lost productivity or physical and mental health, have been surveyed. Burton et al. (48) investigated presenteeism based on employees’ responses to a health risk appraisal (HRA), which included a modified version of the Work Limitation Questionnaire (WLQ) (48). Taken together, USD is causing a substantial economic burden for a disease that is largely preventable.

## Potential and innovative strategies to prevent urinary stone disease

Protective factors for USD include increased fluid intake, healthy eating habits and physical activity. Drinking >2 Liters of water per day is challenging and may be associated with hyponatremia when hydrochlorothiazide is also prescribed (12). Other strategies are the consumption of citric juices, and restricting animal protein and salt to <2000 mg/day. High-citrate beverages have been proposed to improve the urine chemistry of patients with calcium stones (49). Importantly, dietary calcium intake should not be restricted in the growing child/adolescent, as it leads to increased reabsorption of unbound oxalate in the gut, a risk factor for calcium oxalate stones.

Few randomized clinical trials have assessed which clinical interventions decrease the recurrence of kidney stones, especially in pre-adolescents and AYA. Multi-site studies are needed to help identify how regional variations in USD incidence may be due to different environmental factors such as rising ambient temperatures, use patterns of antibiotics and environmental toxins (e.g., melamine, cyanuric acid, and polyaromatic hydrocarbons) (12).

Although treatment recommendations have been developed in the historical context of USD (12), we have little understanding of the USD process and current risk factors in pediatric patients. Innovative treatment like completely filtering out microplastics such phthalates from drinking water, which have been linked to USD, need to be explored systematically.

### Summary

Understanding modern risk factors/biomarkers of USD, with special attention toward sex differences, and the potential impacts of simple interventions for pre-adolescents and AYA with USD, will inform clinical research and trials, therapeutic considerations, public health interventions as well as policy decisions. Opportunities for intervention exist both by reducing nidus crystals such as monobutylphthalate and modifying the lithogenic milieu of the urine, specifically urinary calcium oxalate supersaturation and other potential crystals (i.e., uric acid).

## Conclusion

There is an unprecedented increase in USD, particularly among children, adolescent, and young adult women. The reasons for this increase remain unknown. This chronic health condition is rapidly changing from one of older men to one of younger women. The trend is perplexing, not only from a health care and economic burden perspective, but also due to the often-recurrent formation of stones in the urinary tract, and the associated long-term co-morbidities such as osteoporosis.

These specific sex differences have been underappreciated, as female USD patients aged 10–35 years have long surpassed their male counterparts. The rate of increase is so vast, that dietary changes such as high salt intake alone may not correct this phenomenon. Our findings from two different countries with different health insurance systems are concerning. Further multi-site investigations will confirm our findings and may help identify risk factors and measures to reverse this concerning trend. The research must investigate environmental, hormonal, metabolic, behavioral, and socio-demographic factors along with financial burden, to identify opportunities for intervention. Appreciating the sex differences in USD may also have a major impact in the therapeutic approach. In view of the sex differences outlined here, treatment strategies such as high-water intake, citrate supplementation and alkalinization of the urine may not be appropriate for both sexes.

## Research in context

### Evidence before this study

Urinary stone disease (USD) is increasing around the world. It used to be a disease of older overweight men owing to lower urine pH and a lower post-prandial absorption of anions. However, the incidence of USD has shifted to women with the fastest increase in adolescents and young adults (AYA). The reasons for this phenomenon are unclear.

### Added value of this study

We describe the changing epidemiological trends and sex differences of USD in the province of Ontario, Canada over a 15-year-period as well as in a single center in North Carolina, United States of America over a 5-year-period. We illustrate the pathophysiological differences in USD between men and women and discuss possible etiologies including microplastics as possible nidus of USD. Microplastics are metabolized by the kidneys and there is a 3-fold difference between women and men with regards to the formation of insoluble mono-butyl-phthalate. We also offer suggestions for future research to understand this perplexing sex difference.

### Implications of this study

The incidence of USD has shifted to more women and younger patients, and in fact may become more prevalent than diabetes mellitus. USD is associated with great morbidity and has the potential for chronic kidney disease later in life. Effective strategies to prevent and treat USD need to be developed. The growing public health problem of USD in younger patients requires research efforts to understand the sex differences and to design interventions.

## Data availability statement

The data analyzed in this study is subject to the following licenses/restrictions: property of the Ontario Institute of Evaluative Sciences. Requests to access these datasets should be directed to sumit.dave@lhsc.on.ca.

## Ethics statement

The studies involving humans were approved by Human Research Ethics of the University of Western Ontario. The studies were conducted in accordance with the local legislation and institutional requirements. The ethics committee/institutional review board waived the requirement of written informed consent for participation from the participants or the participants’ legal guardians/next of kin because it was database research through the Institute of Evaluative Sciences at Western, PI SD.

## Author contributions

GF: Conceptualization, Data curation, Formal analysis, Investigation, Supervision, Writing – original draft, Writing – review & editing. SD: Data curation, Formal analysis, Investigation, Methodology, Supervision, Writing – review & editing. VR: Investigation, Methodology, Supervision, Validation, Writing – review & editing. SR: Methodology, Resources, Supervision, Writing – review & editing. DV: Conceptualization, Methodology, Supervision, Writing – review & editing. JH: Conceptualization, Investigation, Methodology, Project administration, Resources, Writing – original draft, Writing – review & editing. JBj: Methodology, Validation, Writing – review & editing. JBu: Methodology, Writing – original draft, Writing – review & editing, Investigation, Resources, Validation. DG: Methodology, Writing – original draft, Writing – review & editing, Conceptualization, Supervision, Formal analysis. JG: Conceptualization, Supervision, Validation, Writing – review & editing. F-CL: Conceptualization, Supervision, Validation, Writing – review & editing. NJ: Conceptualization, Validation, Writing – review & editing. JM: Validation, Writing – review & editing, Data curation, Formal analysis, Methodology, Supervision. HR: Supervision, Validation, Writing – review & editing, Conceptualization. VB: Supervision, Validation, Writing – review & editing, Resources. PW: Resources, Validation, Writing – review & editing, Investigation. SC: Validation, Writing – review & editing, Methodology, Supervision. NS: Methodology, Supervision, Writing – review & editing, Resources. JD: Resources, Supervision, Writing – review & editing, Validation. LF: Resources, Supervision, Validation, Writing – review & editing. MD-G: Resources, Supervision, Validation, Writing – review & editing, Conceptualization, Data curation, Investigation, Methodology, Project administration, Writing – original draft.
